# Comment on “Optimal Exposure Biomarkers for Nonpersistent Chemicals in Environmental Epidemiology”

**DOI:** 10.1289/ehp.1511057

**Published:** 2016-04-01

**Authors:** Richard W. Stahlhut, Richard B. van Breemen, Roy R. Gerona, Julia A. Taylor, Wade V. Welshons, Frederick S. vom Saal

**Affiliations:** 1 Division of Biological Sciences, University of Missouri, Columbia, Missouri, USA; 2 College of Pharmacy, University of Illinois at Chicago, Chicago, Illinois, USA; 3 Department of Laboratory Medicine, University of California, San Francisco, San Francisco, California, USA; 4 Department of Biomedical Sciences, University of Missouri, Columbia, Missouri, USA

In a recent Brief Communication, Calafat et al. expressed concern that epidemiological studies inappropriately assess exposure to nonpersistent chemicals such as bisphenol A (BPA) and phthalates by measuring chemical concentrations in serum and tissues. They assert that urine is the most scientifically valid matrix and that accurate measurement of other matrices is difficult due to contamination of samples and assays. We believe their assertions require clarification.

The scientifically appropriate matrix is determined by the study objectives. For population studies, we agree urine is an appropriate matrix to initially probe whether exposure to a nonpersistent chemical is associated with a disease or risk factor. However, Calafat et al. appear to target more than population studies. They illustrate the purportedly growing problem of non-urine measurement in epidemiology with a list of 80 studies, cited by PubMed identification numbers (PMIDs), which surprisingly includes pharmacokinetic and experimental studies.

Of these 80 studies, 35 arguably required non-urine matrices to achieve study objectives. For example, in five studies (PMIDs 10716589, 10964036, 11604266, 17661831, 23145999) the subjects were dialysis patients—i.e., people without normal capacity to produce urine. One study used a placenta perfusion system to examine phthalate distribution between maternal and fetal circulation (PMID 17049806). A dog study (PMID 23761051) found unmetabolized BPA was rapidly absorbed into circulation following sublingual administration. A human study (PMID 25337790) exposed participants to BPA-containing thermal receipt paper and found a substantial increase of unmetabolized BPA in serum. It seems inconceivable to us that Calafat et al. would consider such studies inherently flawed.

For chemicals excreted in urine, the urinary concentration provides an estimate of exposure. However, the bioactive form in serum and tissue is what alters physiology. When a nonpersistent chemical is absorbed via the gut, first-pass metabolism by the liver can dramatically reduce the amount of unmetabolized compound reaching the bloodstream as compared with other routes (Søeborg et al. 2014). Therefore, for chemicals in widespread undocumented use—where route-of-exposure information is unavoidably incomplete—one cannot accurately predict the internal concentrations of the unmetabolized compounds with urine measurements and a model that includes only gut absorption. Such models may grossly underestimate internal bioactive dose from non-gut exposures and incorrectly suggest that measurement of higher-than-predicted serum concentrations is due to contamination.

In our view, Calafat et al. suggest that non-urine measurements are invariably contaminated. However, contamination cannot explain the results of the studies by Gayrard et al. (2013) and Hormann et al. (2014), which demonstrated classic pharmacokinetic curves with logical interrelationships between the parent compound and metabolites. Furthermore, the proposition that contamination is unavoidable is contradicted by numerous studies spanning 15 years (vom Saal and Welshons 2014). For example, in a paper coauthored by Calafat (Ye et al. 2013), the authors reported accurately measuring BPA in human serum after identifying and eliminating contamination. Subsequently, Vandenberg et al. (2014) reported a blinded study directed by the National Institutes of Health (NIH) in which several U.S. laboratories accurately measured BPA in human serum spiked by NIH personnel. Arguing that chemical X cannot be measured in tissue Y because of contamination is an odd position to take, given that eliminating sources of contamination is a normal part of the development and validation of any assay—as was clearly described by Ye et al. (2013).

In summary, without further clarification, the Brief Communication by Calafat et al. could easily be interpreted as proposing that human environmental studies of any kind must measure nonpersistent chemicals and metabolites only in urine if they are to be funded and published. Such an interpretation would greatly restrict our ability to move from surface-level exposure measures to internal dose, pharmacokinetics, and *in vivo* pathophysiology. Given the prominence of the authors in environmental health research, this issue needs to be clarified.
